# Focus group discussions on the coverage of the southern separatist movement crisis in Yemeni newspapers

**DOI:** 10.1186/s40064-015-1000-0

**Published:** 2015-05-15

**Authors:** Fatima Mohamed Al-Majdhoub, Azizah Binti Hamzah, Moh Yahya Ariffin

**Affiliations:** Department of Media Studies, University of Malaya, Kuala Lumpur, Malaysia; Faculty of Leadership and Management, Universiti Sains Islam Malaysia, Kuala Lumpur, Malaysia

**Keywords:** Focus groups, People’s perception, Southern separatist movement, Thematic analysis

## Abstract

A qualitative method using focus group discussions (FGDs) was applied in this study to identify people’s perceptions on newspaper reporting pertaining to the Southern Separatist Movement (SSM) by different Yemeni newspapers. This paper also looked into the attitudes towards the movement and the popularity of the issue of Yemeni unification. Five FGD groups with a total of 30 participants discussed the subject and some other aspects related to it. The findings of the focu19 groups showed that the southern crisis and SSM had shaken the people’s trust on the current form of the unity. The discussion with the groups revealed that media in general and the selected four papers from various political persuasions have no credibility and objectivity, but these papers are trying to instill democratic values which is consistent with their ideology, which have a serious impact on the value of liberal democracy. The participants assured that reporting on the southern cause and the SSM indicated the absence of professional journalism in the media and the political discourse in general.

## Introduction

The Yemeni Unity established on 22 May 1990 was based on an agreement and acceptance between the leadership of the two parts of Yemen, Yemen Arab Republic and People's Republic of Yemen to be united into “The Republic of Yemen” with the extension of sovereignty all over the new country. Three years later a conflict began to occur between the partners of the unity which led to the 1994 civil war when the leader of the Yemeni Socialist Party Ali Salem Al-bidh officially declared the first call for secession. The victory of the northern forces in the south had left the southerners with grievances and deteriorating situations.

In 2007 anti-government protests took to the streets demanding the government to solve the poor living standards and discrimination against the southerners since the 1990 unification and the 1994 civil war by the former regime in the north. The increasing complaints of unequal treatment of southern Yemenis by their northern counterparts led to the foundation of the Separatist Southern Movement (SSM) that is known in Yemen as *Al HarakAl-Janubbiy* (Salisbury 2014).

In late 2009 the SSM openly called for secession and reinstating the former independent south Yemeni state due to the irresponsible actions by the government in responding to the protests and the unexpected boost gained by some influential figures that used to be the allies of the former regime in Sana’a. The southern cause in Yemen is considered the key issue discussed at length in the National Dialogue Conference (NDC). NDC was held after the Yemeni uprising in 2011, which was attended by all social and political players backed by the Gulf Cooperation Council. This national event in Yemen resulted in dividing Yemen into multi-regions and formed the Federal Republic of Yemen.

### The evolution of the southern separatist movement

South of Yemen Movement, also known as the Southern Separatist Movement (SSM) or *Al-Harak Al-Enfasli Al-Janubbiy* (in Arabic) is a term given to a popular movement active in the South of Yemen since 2007. This movement started when former southern military officials and other civil employees forced into early retirement demanded for better pensions. The government responded with oppressive methods such as pursuing, arresting and bribing the leaders of the South. Due to the implementation of these methods, the movement accelerated to an armed struggle pushing for secession from the north. In 2010, the SSM was shown to have the support of 70 % of the southern population and it is expected to further increase (*Yemen Times*, November 6, 2012).

According to Horton (2011) The Southern Movement in the South of Yemen is an umbrella organization for many secessionist-oriented groups in the South. These groups utilized the increasing weakness of Ali Abduallah Saleh’s regime, the heavy handedness of his government in responding to the anti-government demonstrations, and the absence of central state authority in many parts of the south to provide SSM and its member groups more political capital and more opportunities to assert authority over the affairs of southern Yemen.

In the beginning the media did not pay attention to the movement and the southern crisis due to two reasons. First the former regime denied it and promoted that these groups protest to increase their pensions. Second the absence of a single leadership for the Southern Movement weakened the stance of the movement inside and outside Yemen. When the tension escalated throughout the south sparking the fears of another civil war, the media outlets started to heavily cover the events in the south, which resulted in banning of some newspapers and the arrests of journalists in 2009.

The primary point is that the public media have located the movement and the unification of the country at the core of their discourse. More spaces of media coverage were given in radio and TV channels as well as the print and Internet press. The narrative heavily promoted and emphasized on unity as the only source of strength, dignity and independence (Abduh, 2010). Furthermore, there was an exchange in portraying the actions between the southerners and northerners. Public media described the activists of the Southern Movement as a ‘bunch of mercenaries’, ‘self-interested collaborators’, and traitors’, while the southerners portrayed the acts of the north as an occupation. For the southerners, the civil war reminded them of what happened when Iraq invaded Kuwait in 1990 (Boucek, 2009; Dahmas 2009; Aludani, 2010), therefore the protesters chanted slogans that were used during the fight against the British, ‘out, out occupation’.

Abduh (2010) examined the popularity of the National Unity in the official political discourse and assumed that it is getting lesser attention not only in the south but in the whole country. His assumption was supported by an online public opinion poll published on an independent national news website that asked the respondents whether the official political discourse for Yemeni unity enjoyed the same popularity as it was in 1990 or unity after the1994 war. Among 867 respondents, 87.31 % responded with ‘No’, 11.53 % ‘Yes’ and 1.15 % ‘Don’t know’ (Subject for voting, Al-Tagheer, n.d.).

In critical times such as during crises or conflicts, the media are expected to play key roles in portraying true events in order to reflect the reality of the narratives. In doing so, the main purpose is to influence people’s minds in terms of either shaping or changing the public opinion and attitudes toward certain issues. That is because the media are the main sources of information for the public to know what is happening, however the question of the efficiency and professionalism of the media on reporting keeps the door open for more debates.

### Objectives and significance of the study

This aim of this study is to identify people’s perceptions on media coverage of the SSM, as well as their perceptions on the movement and the popularity of the idea of Yemeni Unification. Using a thematic analysis, this study asked the following questions:

1. What perception do people have about the SSM and the popularity of the Yemeni Unification?
2. What do people think of the coverage of the SSM by the different newspapers?

The findings of this study were able to provide useful information about the SSM, the efficiency of the Yemeni media and other related aspects objectively from the Yemeni people’s point of view. There is a lack of public opinion studies on the efficiency of the media from different political parties, or how the media operate in Yemen. Therefore, this study is considered to be significant in providing recommendations on how the Yemeni media should operate during the time of crises particularly the partisan media in order to contribute to ethical media reporting and politics.

### Theoretical thematic analysis

In this paper, we believe that the thematic analysis offers an accessible and theoretically-flexible approach to analysing qualitative data. Braun and Clarke (2006) discussed theory and method for thematic analysis, and managed to clarify the similarities and differences between different approaches that share features in common with a thematic approach. They concluded that thematic analysis is a useful method for qualitative research in and beyond psychology. Thematic analysis is a method of identifying, analysing and reporting themes within data; it also helps in interpreting various aspects of the research topic (Boyatzis, 1998; Braun and Clarke 2006).

Qualitative research in social sciences refers to textual data generated from in-depth interviews and focus groups which are often transcribed precisely from audio recordings and participant observation notes. And, in the vast majority of cases, thematic analyses, rather than word-based approaches, are used for that because it is still most useful in capturing the complexities of meaning within a textual data set and it is also the most commonly used method of analysis in qualitative research (Guest et al. 2011).

### Samples and methods

To examine people’s perceptions on the coverage of the SSM by the Yemeni newspapers, four different newspapers were selected purposively to serve the objectives of the study. The newspapers were: one official newspaper which represented the government (*Al-Thawrah*), two partisan newspapers that represented the two strongest opposition parties *Islah* Party (*Al-Sahwah)* and the Yemeni Socialist Party *(Al-Thawri*), and one independent newspaper (*Akhbar Al-Youm*). These newspapers were accessible in their archives and the researchers were also able to easily acquire hard copies for the purpose of this study.

### Content analysis: samples

Before the researchers proceeded with the focus groups, the content of random samples of the four newspapers were analyzed in order to identify how these different newspapers presented or framed the SSM. The time frame of analyzing the content of these four newspapers was from January to December of 2009. This period is chosen because it is considered as the point when SSM reached its peak. This study adopted the five news frames that are commonly used in media discourse, known as the ‘Generic frames’ or ‘Structural themes. These areas consist of the conflict, human interests, responsibility, consequences, and morality frames (Semetko and Valkenburg 1999; Matthes and Kohring 2008).

It was ascertained that the conflict and responsibility frames dominated most of the articles if not all by the four newspapers. The southern movement and their leaders were severely criticized in calling for secession and spreading violence and hatred among the Yemeni brothers. For example, the leaders of the southern movement were described as “traitors” and “seditious figures” that will never succeed in splitting Yemen (*Al-Thawrah,* 30 April 2009). The ruling party was blamed in escalating the situation in the south, the former president Saleh was criticized in the way he addressed the southern leaders, his denial of the southern cause and the threats in his speeches in a news item entitled “Yemen is fine and what is happening in the south is just a storm in a teacup” *(Akhbar Al-Youm,* 22 May 2009).

Many articles also criticized the way the government chose methods that were utilized to silence the south either by violence or other means. For instance, a writer of an opinion piece described the regime as having dry mind when the regime announced that the capital city would be transferred to the south (Aden) in order to silence the southern movement (*Al-Sahwah,* 23 April 2009). In regards to this, another writer of an opinion piece mentioned that the regime failed to recognize the demands and grievances, and the feelings of injustice felt by the southern people.

The partisan newspapers severely criticized the presidential speech, for example, a writer of an opinion piece article attacked Saleh by saying that he had used inappropriate words to describe the southern movement as actions by secessionists and traitors. The writer questioned Saleh by asking, “How can you accuse the south of being separatists while the fact is, there is no legitimacy of the unity after it had been violated by the regime in 1994?” He added that the persons who should be blamed are from the ruling party and its patronage system that regarded the south as valuable stolen goods or that a piece of land must be returned to its origin in the North (*Al-Thawri,* January 1 2009).

In this regard, Sabah in her study (Sabah 2010) found that the Yemeni newspapers differed in their reporting on internal political issues due to the variation of their political trends and ideologies which in a way generated different news frames.

The researchers noticed that there were no reporting on the deeply rooted causes for the south cause or SSM and the crucial point was that if there was no accuracy or balance in reporting such events, and if the media organizations controlled such information by preventing many stories from being told, then the situation may influence how the public understand, interpret and make judgments of the events. As we will show, in order to establish how the media coverage on SSM is received by Yemeni people, it is necessary to work closer with them for deeper understanding.

### Focus group discussions

To assist in the collection of the essential data, the qualitative method of focus group discussions (FGDs) is applied in this research. This technique is used for raising views and opinions of respondents on certain issues, for example, political issues (Kumar 1987). The focus group discussions can be used to obtain insights into target audience perceptions, reactions, needs, problems, feelings, beliefs and reasons for certain stands and practices; unlike survey questionnaires that ask for responses expressed on five point rating scales or other constrained categories (Khan and Menderson, 1992;Stewart et al. 2007; Krueger and Casey, 2009; Eliot, 2010). There the FGDs provided the researchers with richer data.

Five groups were conducted in Yemen for this study; each group consisted of six participants, a total number of 30 respondents from Sana’a University. The rational of the focus group discussion method was explained to the respondents; more specifically the objectives of the study were clarified during the introduction sessions.

The focus groups were conducted in Arabic and lasted approximately three hours (see Table 1). In this study, respondents carry the identity based on the number of the group, regional (north and south), and the individual number. For example G1N1 indicated respondents from group one, the north part and carried number one, G5S12 indicated respondent number twelve from the south part in group five.

**Table 1:** Focus group discussion

Group no	Participants	Date	Time	Place
1	6 from the North part	27 August 2012	4 pm-7 pm	*Bader* Org.
2	6 from the North part	28 August 2012	4 pm-7 pm	*Bader* Org.
3	6 from the South Part	29 August 2012	3 pm-6 pm	*Bader* Org.
4	6 from the South Part	1 September 2012	4 pm-7 pm	*Bader* Org.
5	6 Sothern + Northern	2 September 2012	4 pm-7 pm	*Bader* Org.

### Focus group discussion

There were 30 respondents in the focus groups; 15 were southerners and the other 15 respondents were northerners. Table 2 shows the governorate of the respondents.

**Table 2:** The respondents’ governorate

Governorate	No. of respondents
Sana’a (N)	9
Aden (S)	9
Tazi (N)	4
Hadramout (S)	4
Ad Dali (S)	2
Raeemah (N)	1
Abyan (S)	1
Lahj (S)	1
Damar (N)	1

Table 3 shows that a majority of the focus group respondents were independent and not politically affiliated to any of the political parties. For those who were politically affiliated, three participants were affiliated to the Yemeni Socialist Party and the other two were affiliated to the General People Congress (The Ruling Party). Under the category, “others” three answered that they are politically affiliated to the SSM / *Al-Harak Al-Janubbiy.*

**Table 3:** Political affiliation

Political affiliation	Participants	Percentage
Politically affiliated	5	16.7
Independent	18	60.0
Others	7	23.3

### Findings

The responses and the discussion in the focus groups generated the following categories with elaboration below.

### Description of the SSM

Responses to the question “Do you agree with the following definition for the southern crisis” as listed in Table 4.

**Table 4:** Definition of SSM

Level of agreement		Frequency	Percentage
	Strongly disagree	3	10.0
	Disagree	6	20.0
	Agree	14	46.7
	Strongly agree	7	23.3

“The Southern issue is the issue of land and wealth that were seized, the issue of identity and history which were wiped out by the north, it is the issue of the right of the South’s people in the struggle for independence and the restoration of their former independent state and its sovereignty over all its territory” (Alduraimeen 2012).

The fifteen participants from the south answered between “agree “and “strongly agree” while the responses of the other fifteen participants from the north are distributed among the categories. The participants were generally responsive when asked to provide a brief definition or description of the southern crisis or issue as an entry to the discussion, and to know their own personal frames about the topic under consideration before they were exposed to randomly selected articles from the four newspapers. Some of the participants had a consensus that the southern crisis is a matter of several issues such as rights, identity and justice, which were taken from the South after Yemeni civil war. These are some of the representative responses:

“*The South is stolen in every single aspect*” (G1N1).

“*The Southern issue reveals the marginalization, identity issue, violation of peoples’ rights, neglecting the legal partnership of the Southerners according to unity’s deal*” (G2N10).

### Priority of the southern crisis

Focus group participants were asked to determine the priority level of the southern cause since the country was facing other crises at the same time, Table 5 indicated the result.

**Table 5:** The priority of the southern crisis

Priority of the crisis		Frequency	Percentage
	Low priority	3	10.0
	Neutral	6	20.0
	High priority	21	70.0

Most of the focus group participants from both the south and the north positively responded that the southern crisis is a high priority issue when they were asked “Do you think the southern crisis is high priority among the other crises facing the country? For example, respondent G5N15 said in a sad tone:

“*You may see the consequences of this crisis on social fabric…it has produced hatred, discrimination and regionalism among the brothers to a degree that innocent people from the north were killed in the south due to the wrong priming either from the media or the leaders of the Southern Movement”.*

Generally, many participants from the south and the north in the focus groups believed that this crisis must be classified as the most important and of high priority since it threatened the unification of the country and affected the harmony of the Yemeni social fabric. However, there were three participants who stated that, the southern crisis is not considered a priority crisis since the country is facing other pressing crises such as the *Houthi* rebellion and the existence of al-Qaeda on Yemeni soil, besides the economic, health and education problems which should receive more attention.

### Sources of information

The media as well as interactions with the southern people were the sources of information that most of the focus group participants attained their information about the southern crisis and *Al-Harak Al-Janubbiy*. One of the northern participants G1N1 stated that he did not get his information from the above sources but from the YSP as he politically affiliates with that party. Other participants G2N9 and G2N12 said they did not know what the southern crisis was until the Yemeni uprising in 2011 erupted and interacted with the activists of the Southern Movement in change square in Sana’a where all anti-government protesters gathered. The respondent G1N5 said that the way the media presented the southern crisis; more specifically the official media led him to seek other trusted sources of information. While several participants G3S1, G3S2, G3S3, G4S11, G5S15, G3S6 and G3S5 from the south said that they received their information from the independent newspaper *Al-Ayyam* which was banned in 2009.

### Knowledge about the causes of the southern crisis

When the participants were asked, “What are the causes of the southern crisis?” many related their opinions with more details, such as stating the fact that the main cause was the political, economic and social marginalization of the south and the absence of equity between the north and the south. Specific answers claimed that the causes were the oppression over the south; the issues of the military officials who were forced into early retirement and the civil workers who were dismissed from their jobs without respecting their rights. In addition, other causes include exploiting the economic resources of the south and looting the lands by some elites of the previous regime after the civil war in 1994. The respondent G1N4 affirmed by saying:

*“I think the media play some part in these causes. The misreporting and biased coverage of the media in the south have incited against anything north, while the official media did neglect the crisis when it first started. But when the pressure reached a higher level by the movement, they started describing the southern people as traitors and targeting the National Unity”.*

### Agreement on the legitimacy of the SSM

All of the focus group participants from the south and some from the north responded positively when asked “Do you believe in the legitimacy of the southern issue and the Southern Movement” (Table 6).

**Table 6:** Legitimacy of the southern cause

Belief in SM’s legitimacy		Frequency	Percentage
	Do not believe	4	13.3
	Believe	26	86.7
	Undecided	0	0.00

The legitimacy of social movements has been debated in several researches. How the movements gain their legitimacy is argued to be based on the sources of the legitimacy. Lis (2011) concluded that there are four categories that can determine the legitimacy of movement, which are legitimacy of goals, numbers of supporters, and ability of the movement to convince its rights by using appropriate means, and the promised result or outcomes.

Based on the aforementioned presentation, the focus group participants were asked to express their opinions about the legitimacy of the SSM based on the goals and the outcomes of the movement. The participants were also asked about the legitimacy of the characteristics of the Southern Movement’s leaders.

G4S11 said:

*“Since the southern issue and Al- Harak Al-Janubbyi demand separation after a long time of being patient…waiting and addressing the grievances and condemning the wrong policies over the partner of the unity’s agreement… as everyone in the south believes that we have rights… we asked for equality at the first stage by using civilized means… but no one had listened, so we tried other means to mobilize and to make our voices heard …either we have been heard or not … the southerners represent legitimate independent country…therefore from this point I can tell you that both the issue and the movement are legitimate as long as they represent us ‘southern people”.*

### Attitudes of the participants

Responses to the question “What is your attitude towards the southern issue?” are listed in the Table 7.

**Table 7:** Attitude towards the southern cause

Participants’ attitude		Frequency	Percentage
	Unfavorable	4	13.3
	Neutral	9	30.0
	Favor	15	50.0
	Undecided	2	6.7

Answers to the question on “What is your attitude towards the SSM/ *Al-Harak Al-Janubbiy?*” are listed in the Table 8.

**Table 8:** Attitude towards SSM /Al-Harak Al-Janubbiy

Attitude towards *Al-Harak Al-Janubbiy*		Frequency	Percentage
	Unfavorable	6	20.0
	Neutral	14	46.7
	Favor	9	30.0
	Undecided	1	3.3

Half of the focus group participants chose ‘Favor’ when asked about their attitude towards the southern issue. The participants were more likely to have consensus that the southern issue is all about the unjust treatment to the south after the 1994 Yemeni civil war, and the grievances which could have been solved fairly without cracking down the peaceful movement. When the participants were asked about their attitudes towards the Southern Movement, 46.7 % were neutral, 30 % favored and 20 % unfavorable.

During the discussion some of the participants provided more information about the Southern Movement /*Al-Harak Al-Janubbiy* when they were asked, “who brought the southern crisis/ issue to the surface and what is actually the Southern Movement?”, some participants stated that the Southern Movement/*Al-Harak Al-Janubbiy* brought the southern issue after the civil war; therefore it is the mouthpiece of the southerners.

The respondent G3S2 said

*“The Harak Al-Janubbiy consists of the southern people… injecting the Southern Movement with some of the armed people does not mean that the whole Harak is bad… Al-Harak Al-Janubbiy is a component of the southern people who have the right to express and restore our land and identity… The unity has destroyed and marginalized the leadership of the south that resulted in the disagreement among the leaders of the Southern Movement… we are not going to lose hope that the south is able to generate new leadership and rebuild civil institutions in case we restore our country”.*

In the focus groups there were some participants who were totally against the southern issue and the Southern Movement, for example the respondent G1N6 confidently said:

*“I do not support either the southern issue or the Southern Movement…the problems that the southerners argue about are also here in the north…oppression, lands are taken and grievances… if every group in Yemen follows what the Southern Movement has done, we can say “goodbye” to our country… besides some of the southern politicians intentionally incite against everything northern… the killing crimes committed in cold blood on innocent northern people living in the South proved the reality”*

### Impact of the southern crisis on the popularity of Yemeni national unity

About 53.3 % of the participants agreed that the southern issue has affected negatively on the popularity of the Yemeni national unity when they were asked, “Do you think the SSM has affected the popularity of the Yemeni Unification? (Table 9).

**Table 9:** Impact of the SSM on the Yemeni unification

Level of agreement		Frequency	Percentage
	Strongly disagree	2	6.7
	Disagree	3	10.0
	Agree	16	53.3
	Strongly agree	9	30.0

When the participants were asked “Do you think the SSM has affected the popularity of the Yemeni Unification, about 53.3 % participants agreed that the southern issue has affected negatively the popularity of the Yemeni national unity while 30 % “strongly agree”.

A few participants agreed with what respondent G4S7 said, when he stated:

*“What could be worse than killing citizens by their identity…? How do you expect a unity to last in this atmosphere? The southern issue affected negatively on the way the new generation think about the unity which is associated with regional discrimination, spreading hatred between the southerners and northerners…definitely if there is voting on the unity now, it is not likely to reach 30 %”.*

In more challenging and aggressive manner, the respondent G3S1 said:

“*The southern issue is the light for us ‘southerners’ …Yes; we should thank the Southern Movement in revealing the truth about the unity to the southerners … Since the 80s “we” the South had fought and struggled for the unity and now we are fighting to restore our independent country… we have taught our children that their rights have been taken by the north and that they should fight for it…the civil war in 1994 had murdered the Yemeni national unity and I can predict that more than 86 % of the unity is declining in the south…besides the northerners now are not welcomed in the South”.*

### Importance of the Unity

When the participants were asked if they believe in the importance of the Yemeni unity, 76.7 % reported that it is important while 20 % think that the unity is not important and 3.3 % could not decide. Table 10 shows the result.

**Table 10:** The Importance of the Yemeni unification

Level of importance		Frequency	Percentage
	Not important	6	20.0
	Neutral	1	3.3
	Important	23	76.7

Surprisingly, 76.7 % of the focus group participants agreed on the importance of the Yemeni unity when asked “Do you think the Yemeni unity is important?”, while 20 % said it is not important. Those undecided or neutral is 3.3 %. It is known that the northern society is more religious and tribal and the unity has gained its value from these thoughts. The unexpected notion was that out of six who said the unity is not important, three participants were from the north. G1N1, G2N10 and G2N11 argued that unity is not a religious obligation or among the pillars of Islam”. For example G1N1 said “There is no need for the unity after the 1994 civil war”.

Another respondent G2N10 assured by saying:

*“I do believe in the unity as a concept but I do not believe in the importance of Yemeni unity …in fact the Yemeni people before the unity were one nation in two different countries, but now two nations in one country…the Yemeni unity has changed the valuable meaning of the concept unity to “unity is a red line” and “unity or death”*

### The southern crisis is a real threat

Table 11 lists the responses to the question “Do you agree with the statement which says that the southern issue is a real threat to the Yemeni national unity in particular and the security and stability in the region in general?”

**Table 11:** The southern crisis threatens the unity and region

Level of agreement		Frequency	Percentage
	Strongly disagree	4	13.3
	Disagree	4	13.3
	Agree	16	53.3
	Strongly agree	5	16.7
	Undecided	0	0.00
Missing case		1	3.3

Most of the focus group participants strongly agree that the southern crisis is considered a real threat to the security and stability of Yemen in particular and the region in general. Here are some of the common responses,

G5S14 said:

“*The issue is a threat to the stability of the country …it is obvious from what we can see in the daily events in the South…you cannot even walk in the street in peace especially in the governorates where the government’s control is weak… some groups have utilized the conflict between the government and the Southern Movement to implement their agendas, especially the various gangs, al-Qaeda militants or other groups”.*

A few participants mentioned that the crimes that were committed by the Southern Movement are proof that the issue threatens public peace. G1N6 said:

*“The crimes of Mala’ah and Habeel Jaber were targeted in cold blood, the northerners have brought sadness and terrifying memories to what happened in 1986 when people were murdered by their ID, what could be more of a threat than this!!”*

### Exposure to the newspapers

Table 12 shows that majority of the focus group participants do expose themselves to the four selected newspapers or read them on a daily basis (60 %). However, *Al-Thawrah* scored (16.7 %), none of the above (13.3 %), *Al-Thawri (*6.7 %), and *Akhbar Al-Youm* (3.3 %).

**Table 12:** Exposure to the newspapers

Newspaper	Frequency	Percentage
*Al-Thawrah*	5	16.7
*Al-Thawri*	2	6.7
*Al-Sahwah*	0	0.00
*Akhbar Al-Youm*	1	3.3
All together	18	60.0
None of the above	4	13.3

To meet one of the objectives of this study which is identifying people’s perceptions on the coverage of the SSM and whether the participants do read these newspapers on daily basis or not, the researchers make sure that the participants give their opinion based on actual reading. Therefore during the applicable sessions the participants were asked to randomly select articles from the four newspapers and read for about 30 min followed by discussion. Some of the participants’ opinions are mentioned as follows:

G4S9 *“We “southerners” dislike Al-Sahwah newspaper and Islah Party… we do not read the Islamic newspaper due to its attitudes towards the southerners. G5S14 mentioned “Al-Zandani a member of Islah Party issued a fatwa- a ruling on a point of Islamic law given by a recognized authority-during the civil war in 1994 to destroy and kill the southerners because they are “infidels” …we were treated like chickens!”*

G4S9 claimed and supported by G4S11 when she said, “*The unity was rejected from the north especially from the Islamists wing such as Al-Zandani who issued fatwa to reject the unity with “communists” … we have been labeled as “disbelievers”. I live in Sana’a but am still this ‘Adeni’ girl with no Adabb –immoral-… I do not need to read that in their newspapers either”.*

### The perceived influence of reporting

Table 13 indicates that 86.7 % of the focus group participants were not influenced by the way the newspapers reported the southern crisis and 13.3 % were slightly influenced when they were asked “Have your perceptions been influenced by the way these newspapers report the southern issue and the SSM/*Al-Harak?*

**Table 13:** The perceived influence of reporting

Level of influence		Frequency	Percentage
	Not at all influenced	26	86.7
	Slightly influenced	4	13.3
	Somewhat influenced	0	0.00
	Very influenced	0	0.00
	Extremely influenced	0	0.00

Surprisingly, all participants of the focus groups expressed that there is no credibility and objectivity in reporting the southern crisis and *Al-Harak*. Here are some of the responses, G1N2 said:

*“None of these newspapers have affected my personal frames about the issue or Al- Harak…all we know that these papers are the mouthpieces of the owners, either opposition parties or the regime… that means they have to disseminate their political agenda and ideologies towards the issues based on their interests”.*

Almost all the participants pointed at the credibility and objectivity of reporting the southern issue and movement by the newspapers. For instance, G5N11 said: *“None of these newspapers framed the southern issue or the Southern Movement neutrally; these papers have no transparency, credibility and objectivity… I think that is impossible”.*

Other participants said that these newspapers are the representatives of their owners and ideologies, G2N8 said “I have read the articles from the four papers and I think these papers have reported the issue from the microscope of the owners. We all know that these papers are the mouthpieces of the regime and opposition parties, so when they report any issue, that would be based on their ideologies and political interests*”.*

The participants expressed more by giving examples. G2N8 further explained:

*“Al-Thawrah the official paper never reported the southern issue and the daily events in the South … instead by using improper language described the Southern Movement /Al-Harak as traitors, mercenaries and outlaw groups … devoted more articles about the national unity as a holy figure, its achievements… and the red line should not be crossed… besides sending warning messages or threats to use force upon whoever tried to abuse the unity…Yemen will be another Somalia… it is like enforcing the unity”.*

G3S6 stated:

*“Al-Sahwah is the voice of the biggest opposition party Islah … before 2009 used to report the issue in a way to provoke the regime because it was their conflict season with the ruling party… and after 2009 their political attitude towards the southern issue changed because of its disagreement with YSP… And I do not think these papers have any influence on the public perceptions… simply because they are not trusted ones, no accuracy or balance in reporting”*

G5N13 agreed with G5S13 when the latter said *“Unfortunately, these newspapers have proved that there is professional journalism in Yemen”.*

### Slogans

Responses to the question “What do you think of the acceptance of the slogans “Unity or death” “Secession or deaths” are listed in Table 14.

**Table 14:** Acceptance of slogans

Level of acceptance		Frequency	Percentage
	Unacceptable	26	86.7
	Neutral	0	0.00
	Acceptable	4	13.3

About 86.7 % of the focus groups responded “unacceptable” when asked “Do you accept the slogans “unity or death, separation or death?”. G4S10 said *“The use of the slogan “the unity or death” appears many times in the newspapers. It is like “Do not ever think of separation otherwise death would be your fate… It is sort of reminder for what happened in 1994”.*

For those who accepted the use of the slogans, the respondent G1N6 challenged by saying *“Wa Allah” -I Swear to God”- even if we would lose every single drop of our blood for unity…no separation will take place”.* Another respondent from the South said “*Well, if the northerners insist in using this slogan “Unity or death”, we “southerners” do not mind dying for separation*” (G5S15).

### Attitude towards secession

Table 15 shows the answers of the focus group participants on the question “Do you support/ oppose the secession as demanded by the Southern Separatist Movement?”

**Table 15:** Attitude towards secession by SSM

Attitudes of the participants		Frequency	Percentage
	Unfavorable	22	73.3
	Neutral	0	0.00
	Favor	8	26.7

About 73.3 % of the focus groups participants chose ‘oppose’ in solving the southern issue when asked, “What is your attitude towards separation?”Many participants mentioned that the unification is not the problem. Here are some of what have been stated.

G1N3 emphasized that *“What I have seen in the south …the majority of the south does not support the separation especially these days in Aden… and I am sure even if there is self-determination the unity will last*”.

About 28.7 % of the focus group participants ‘favored’ the separation. For instance, the respondent G3S2 said:

*“The southerners have the right to secede, because we had given up our independent county, sovereignty and flag… since the unity did not work out as we hoped, so we have the right to secede according to the unity’s conditions which were changed after the civil war”.*

### Attitude towards the right for self-determination

Table 16 shows the responses of the focus group participants on the question “Do you support/ oppose the self-determination as demanded by the Southern Movement?”

**Table 16:** Attitude towards the right for self-determination

Attitudes of the participants		Frequency	Percentage
	Unfavorable	5	16.7
	Neutral	3	10.0
	Favor	22	73.3

Most of the participants ‘favored’ the right for self-determination when asked “What do you think of the right for self-determination?” For example, G1N4 said:

*“It is the time for Yemenis to decide their future by themselves neither the GPC nor the YSP or other groups… we are not going to repeat the same mistake in 1990 for not voting for the unity project … but since the southern issue is strongly disputable… giving the right to the Yemeni people for self-determination will be the right solution and everyone should be responsible for their choice”. G2N12 said: “I think of self-determination as either we go for the unity or separation, but it will be the right decision because it is all about the peoples’ acceptance”.*

## Discussion

The focus groups that were conducted presented an opportunity to know people’s perceptions and feelings on the southern crisis “which is mostly addressed as the southern cause” and generated the so called *Al-Harak.* The movement was formed in 2007 and threatens the Yemeni unity by openly calling for secession. Although these groups were not representative of all segments of the Yemeni society, opinions obtained provide information on what people think of reporting the Southern Separatist Movement crisis and the Yemeni unity by different Yemeni print media. In addition, for more understanding these groups highlighted other related aspects to the topic in hand during the discussion. The information obtained from the focus groups has shown that the SSM crisis represents the key issue, and real threat for Yemenis to address since the pro-independent sentiment is on the rise. According to the participants the newspapers did not reflect what was happening on the ground. Day (2010) stated that dealing with the Southern Movement simply as security threat, without addressing the underlying political problems that gave rise to it, could become a self-fulfilling prophecy.

Dahmas (2009) stated that the southern crisis worsened when many different groups utilized it for their political concerns and conflict over power which caused confusion about what and who is really *Al-Harak.* The other factor that makes *Al-Harak* inconceivable is the use of regionalism and hatred as weapons, which were translated in the actions of some of the *Harak*’s elements in killing and attacking the northerners living in the South.

Participants in focus groups have stated that they do read the four newspapers but not as trusted sources compared with the trusted papers such as *Al-Ayyam*. They mentioned that somehow they read them to see how the political parties tend to get their political message across the public on issues more specifically toward the southern crisis. The participants provided identical reasons for not trusting these newspapers in regards to the southern crisis and the way of reporting the Southern Movement/*Al-Harak Al-Janubbiy*. Participants stated that these newspapers have no credibility and objectivity. They further said that there is an obvious lack of professionalism in the treatment of the content of the newspapers. According to them, the newspapers did not provide news analysis or investigative reports. Instead, they only reflected the opinions of the leadership of the political parties.

Overall, these focus groups expressed that there is a cultural gap between the southerners and northerners; unfortunately, the media increase it by their biasness in presenting the southerners and their cause. One of the participants in full rage mentioned that the “These newspapers that I have read during the applicable session have shown their hideousness and lack of journalistic professionalism… and no wonder there is no future for journalism in Yemen”. In this respect, Al-Danani (2008) found in his study of journalism trends in print media that journalists believe Yemeni media have a long way to go before being considered a true public opinion power. He also indicated that there is not much respect for print journalism due to lack of professionalism and unethical behavior by some journalists who neglect their responsibilities and duties and seek other means of income because of the poor economic conditions.

The findings of the focus groups indicate that the partisanship and the ownership of the media shape the news on the southern crisis without objectivity in searching for the facts of this political conflict. Participants proved during the session by analyzing some news items that different newspapers wrote the story about the southern crisis based on the political orientation of the paper, which derived from its ideology. For instance, some pointed at the contradiction in *Al-Thawrah* newspaper as the mouthpiece of the regime. They said on the one hand; the newspaper denied the existence of the southern issue although the violent events reached its peak in the South. In this regard, a number of news items were repeating the statements of the former president, “Yemen is fine and there is no fear on the unification” and “What is happening in the South is just a storm in a teacup”. Besides, news items addressed the Southern Movement with offensive words such as “bunch of mercenaries, traitors, evil people, terrorists etc.” On the other hand, the regime directed a presidential committee to investigate the issue of lands in the South as well as forming popular committees for protecting the national unity since the attitudes of political powers started to favor the Southern Movement due to the disagreement with the regime.

The participants mentioned, surprisingly, that the coverage of the southern issue by *Al-Thawri*, the mouthpiece of the YSP and the expected promoter of the southern cause sounded very weak in their coverage. According to the participants, *Al-Thawri* reported the issue like any other ordinary daily events. They opined that the newspaper toned down reporting due to the internal fragmentation among the members of the YSP as most of them joined the Southern Movement and formed the radical wing, which demands secession.

The study unexpectedly found disagreement among the northerners themselves and the southerners in regards to the southern crisis and the national unity. During the sessions with the northerners there were even some who were aggressive toward anyone who favors or supports *Al-Harak* or criticized the national unity, and the same situation was found among the southerners for anyone who criticized *Al-Harak* or did not support the southern issue. That certain behavior during the northerners’ sessions can be explained from a religious aspect since calling for separation and contempt for the unity is against what Allah orders. The behavior of the southerners to each other can be understood as some of the participants were strong supporters or in other words affiliated to *Al-Harak Al-Janubbiy* and they were from the cities which started the eruption, and they also come from different fractions of *Al-Harak* which obviously could not reach any single agreement.

In conclusion, the discussion with the groups revealed that media in general and the selected papers from various political persuasions are trying to instill democratic values which is consistent with their ideology, and which presents a serious impact on the value of liberal democracy. Another point worthy of mention is the absence of professional journalism in the media political discourse in general and the Yemeni partisan press in particular. That is manifested through the altercations among these newspapers, which in a way created feelings of hatred for the party and its press and cadres among the members of the society. The lack of objectivity and credibility has led the public to distrust and avoid these newspapers, and shift to information either from the trusted independent, Arab or international presses.

Therefore, this study suggests that for the future of Yemeni journalism, it is necessary to free the press from partisanship and ownership restrictions that limit its freedom, which guarantee practicing journalism without the influence of political parties or powers in order to show the progress of democracy in media messages. In addition, there is further need to conduct research that pay more attention to the areas of conflict and resolve issues for the good of all the Yemenis of all political affiliations.

## Abbreviations

FGD: Focus group discussion
G1S: Group one from the south
G2S: Group two from the south
G3S: Group three from the south
G4S: Group four from the south
G5S: Group five from the south
G1N: Group one from the north
G2N: Group two from the north
G3N: Group three from the north
G4N: Group four from the north
G5N: Group five from the north
NDC: National dialogue conference
SSM: Southern separatist movement
